# Is COVID-19 changing psychiatry?

**DOI:** 10.1192/bjb.2020.103

**Published:** 2020-09-03

**Authors:** David M. Foreman

**Affiliations:** Affiliate Senior Lecturer, King's College London, Institute of Psychiatry, Psychology & Neuroscience, UK

**Keywords:** Primary care, epidemiology, social functioning, COVID-19, diagnosis

## Abstract

Two articles on the potential impact of the current coronavirus pandemic on psychiatry reveal agreement on many points, but opposing positions on the methodology, philosophy and politics of psychiatry's response. This points to the need for psychiatry to audit its approach to evidence when agility is required.

All disasters stress-test the societies they impinge upon. The current coronavirus pandemic is no exception. In this issue of *BJPsych Bulletin*, two authors^1,2^ consider the impact the virus might have.

Perhaps surprisingly, there is much agreement. Neither finds dire forecasts of an imminent psychiatric epidemic convincing, although both recognise that there are risks, for some more than others. Both are concerned that the understandable worries associated with COVID-19 may be excessively medicalised and recommend interventions that will limit this. They also implicitly agree that diagnosis is central to psychiatry's identity and validity, focusing on how diagnostic procedures should respond to a change in our average level of fear, though from opposing positions.

Behind the disagreement lies a clash of methodology as well as philosophy and politics. Like the mills of God, orthodox research into diagnostic validity and reliability both improves diagnosis and clarifies its flaws painstakingly but glacially, through peer-reviewed publication and replication. In contrast, networks of critical professionals and patients can both flag difficulties and propose persuasive solutions, using conferences and social media to promote them rapidly as ‘grey literature’, for incorporation into reports from stakeholder organisations. Crises demand rapid responses, and the rapidly rising tide of concern about the quality of academic literature on COVID-19 suggests that our current approach to evidence lacks agility when that is needed. We hope that these articles spark a conversation about how psychiatry should audit its response to the pandemic, so it can learn and improve.

## About the author

**David M. Foreman**, Affiliate Senior Lecturer, King's College London, Institute of Psychiatry, Psychology & Neuroscience, UK.
